# U = U for all: Advancing equity in HIV prevention

**DOI:** 10.1371/journal.pmed.1005090

**Published:** 2026-05-21

**Authors:** Thiago S. Torres, Paula M. Luz

**Affiliations:** Fundação Oswaldo Cruz, Instituto Nacional de Infectologia Evandro Chagas, Rio de Janeiro, Brazil

## Abstract

Suppression of HIV with antiretrovirals eliminates HIV transmission risk, summarized as Undetectable = Untransmittable (U=U). However, U=U literacy remains unevenly understood and shared, and stigmas persist. In this Perspective, Thiago Torres and Paula Luz outline what is needed to improve equity and accuracy in global awareness and education of U=U.

## Teaser

Suppression of HIV with antiretrovirals eliminates HIV transmission risk, summarized as Undetectable = Untransmittable (U = U). However, U = U literacy remains unevenly understood and shared, and stigmas persist. Equitable and accurate awareness of U = U requires culturally tailored interventions, improved provider education, and supportive policy environments beyond biomedical evidence alone.

Advances in antiretroviral therapy (ART) against HIV have contributed to global rates of HIV suppression rising from 40% in 2015 to 73% in 2024 [1,2]. Adherence to ART leads to HIV viral suppression to undetectable levels, with studies demonstrating that sexual transmission risk is zero when viral suppression is sustained [3], producing both individual health benefits and important population-level prevention effects. These effects are reflected in the “Undetectable = Untransmittable” (U = U) message, first coined in 2016, as well as the World Health Organization’s “Zero Risk” policy brief (www.iasociety.org/zero-risk-transmitting-hiv). Awareness, understanding, and acceptance of U = U is associated with health outcomes, including stigma reduction, enhanced sexual intimacy, the alleviation of physiological distress (such as anxiety, shame, and guilt), and increased HIV service engagement [4]. In this sense, U = U links viral suppression to dignity, empowerment, and quality of life. In this Perspective, we examine the persistent barriers to U = U awareness and acceptance across diverse populations and interventions to promote equitable implementation and broader public health impact.

Despite the strength of the evidence, U = U literacy remains highly unequal across regions and population groups. A meta-analysis involving ~227,000 participants indicated that while U = U awareness is high among people living with HIV (PLHIV), it remains moderate among gay and other men who have sex with men (MSM) and low in the general population [5]. A study from Brazil, for instance, showed that 79% of PLHIV perceived U = U as completely accurate (undetectable viral load means no HIV transmission; 4-points Likert scale), contrasted with only 44% of MSM not living with HIV and 17% of the general population [6]. Geographical disparities have also been shown with studies conducted in high-income settings reporting greater U = U awareness and understanding than those conducted in low-income settings [5]. In sub-Saharan Africa, which accounts for more than 60% of the PLHIV globally and where women and girls accounted for ~63% of new HIV diagnoses in 2024 [1], literature suggests limited diffusion of U = U [4]. In a study from Uganda, for instance, men perceived that it was very unlikely that a couple could have different HIV statuses, even if the partner living with HIV was on ART [4]. In a qualitative study among PLHIV from Rwanda, most participants expressed skepticism or reluctance to accept U = U [7].

At the individual level, U = U understanding might also be influenced by social determinants of health, such as income and education. A study from Brazil that included 23,981 sexual and gender diverse participants (21% PLHIV, 72% HIV–negative, and 7% HIV unknown) showed that higher income and education was significantly associated with higher HIV knowledge, and higher HIV knowledge was associated with a higher odds of perceiving U = U as completely accurate [8]. Meanwhile, in a Canadian study, PLHIV reporting lower education and unemployment were less likely to report awareness, acceptance, and positive impacts of U = U in their lives [9]. These results suggest that U = U literacy is not distributed evenly, such that different regions and populations do not benefit from the social and preventive meaning of U = U and remain vulnerable to outdated fears about HIV transmission.

Similarly, although awareness and acceptability of U = U among healthcare providers have increased over time, there is ample evidence to suggest that healthcare providers do not fully understand U = U or remain reluctant to communicate it [4]. Many healthcare providers remain hesitant to share the U = U message, sometimes preferring softer or ambiguous language such as virtually impossible and negligible [10]. A qualitative study in Malawi highlighted that stakeholders expressed uncertainty of how to communicate the message without causing harm, meaning that while they acknowledged the scientific reality of U = U, they feared that patients would misinterpret viral suppression as being cured, leading them to relax, stop taking medication, or start sleeping around and potentially re-infect themselves [11]. Likewise, a qualitative study conducted in the United States and Australia showed that providers engaged with HIV treatment and prevention used ambiguous or inaccurate messaging regarding U = U [12]. These findings reflect persistent paternalism, fear of patient misunderstanding, and misplaced concerns about patient behavior such as engagement in condomless sex. However, when providers withhold or dilute the message, they inadvertently preserve stigma and limit patient autonomy. Provider bias therefore becomes more than a communication problem; it becomes a barrier to empowerment. If U = U is to function as both a clinical principle and a stigma-reduction strategy, providers need structured support to routinely deliver clear non‑stigmatizing U = U messages that are tailored to patients’ knowledge levels and supplemented with visual aids [12]. Provider education is especially important because the credibility of U = U often depends on whether it is introduced as a routine part of HIV care or framed as an exceptional or controversial message. In practice, provider hesitation can delay patient understanding, weaken trust, and reduce the likelihood that U = U will be shared beyond the clinic.

More studies are needed to inform how to best communicate the U = U message and the actual impact of disseminating U = U messaging on behaviors and clinical outcomes [4]. Scaling U = U equity requires interventions tailored for different populations and levels of access. For example, sub-Saharan Africa bears a higher HIV burden across multiple demographics, whereas in the Americas and Western Europe, the epidemic disproportionately affects key populations such as MSM, and education must be tailored accordingly. In South Africa, where 74% of 7.8 million PLHIV have viral suppression [1], a study described a structured co‑creation process of a U = U messaging tool that involved PLHIV, lay counselors, primary‑care clinicians, and representatives from local HIV advocacy organizations [13]. The web‑ and mobile‑based tool called “Undetectable and You” integrated first‑person video testimonials, narrative framing, and simplified explanations of viral suppression grounded in locally validated idioms and explicitly targeted culturally embedded beliefs, HIV‑related stigma, and trust dynamics within the South African primary‑care settings. Though the impact of this campaign is still to be shown, the effort exemplifies how multiple actors need to be actively engaged and trained to compose accurate, culturally relevant information to help disseminate the U = U message within their own networks and strengthen trust in the intervention. Beyond the content, campaign dissemination strategies must target population and providers level gaps: targeted social media ads on platforms for youth, radio spots for low-literacy/rural groups, partnerships with influencers and advocacy networks, posters with QR code linking to educational videos in healthcare facilities and free online U = U training modules for providers. Peer-led education is particularly important for reaching populations that may distrust formal health institutions or have limited contact with specialized HIV services. U = U should also be integrated into LGBTQIAPN-focused and transgender-affirming health services, where culturally competent, nonjudgmental care can improve trust, normalize viral suppression as part of routine care. For broader reach, U = U messaging should be embedded in primary care, sexual health services and in school-based education.

Ultimately, achieving U = U equity will require more than biomedical evidence alone. Although the scientific basis is strong, the reach and impact of the message still depend on social trust, accessible communication, and supportive legal and policy environments. Persistent stigma, unequal access to information, punitive HIV-related laws, and broader violations of LGBTQIAPN+ and women’s rights continue to limit who can fully benefit from U = U [14]. These challenges are compounded by political instability, rising conservatism, and shrinking HIV funding, which weaken prevention systems and narrow the space for rights-based health communication. Closing these gaps will require coordinated action across health systems, community organizations, and policymakers to ensure that U = U is communicated clearly, understood broadly, and supported by laws and services that align with current science.

## Abbreviations

ART: antiretroviral therapy
MSM: men who have sex with men
PLHIV: people living with HIV
U = U: Undetectable = Untransmittable
